# Visceral pleural invasion predict a poor survival among lung adenocarcinoma patients with tumor size ≤ 3cm

**DOI:** 10.18632/oncotarget.16476

**Published:** 2017-03-22

**Authors:** Tianxiang Chen, Jizhuang Luo, Rui Wang, Haiyong Gu, Yu Gu, Qingyuan Huang, Yiyang Wang, Jiajie Zheng, Chang Gu, Xufeng Pan, Jun Yang, Yunhai Yang, Heng Zhao

**Affiliations:** ^1^ Department of Thoracic Surgery, Shanghai Chest Hospital, Shanghai Jiao Tong University, Shanghai, China; ^2^ Department of Radiation Oncology, Shanghai Cancer Hospital, Fudan University, Shanghai, China

**Keywords:** invasive lung adenocarcinoma, non-small cell lung cancer, VPI, stage I

## Abstract

**Introduction:**

The impact of visceral pleural invasion (VPI) on survival remains controversial for patients with early stage non-small cell lung cancer (NSCLC). This study investigated the survival status of VPI among patients with lymph node-negative lung invasive adenocarcinoma smaller than 3cm.

**Methods:**

We retrospectively reviewed 2537 consecutive patients with pathologic stage I lung invasive adenocarcinoma. All patients had received lobectomy and system lymph nodes resection.

Patients were classified into 4 groups according to tumor size and visceral pleural invasion status. Disease-free survival (DFS) and overall survival (OS) were analyzed to evaluate survival difference between these groups.

**Results:**

548 patients with VPI while 1989 patients without VPI were included in this study. For patients with tumor size ≤2cm, patients with VPI had significant worse DFS (HR,4.85; 95% CI, 2.98-7.91; *p* = .000) and OS(HR,3.52; 95% CI, 1.59-7.78; *p* = .002) compared with non-VPI group. For patients with tumor size between 2-3cm, patients with VPI had significant worse DFS (HR, 1.72; 95% CI, 1.16-2.55; *p* = .006) but no significant OS (HR, 1.31; 95% CI, 0.76-2.24; *p* = .330) compared with non-VPI group. For patients with VPI, there were no survival difference between tumor size 2-3cm group and ≤2cm group for both DFS(HR,1.02; 95% CI, 0.65-1.61; *p* = .939) and OS(HR,1.45; 95% CI, 0.71-2.97; *p* = .315).

**Conclusions:**

VPI could predict a poor survival even for node-negative invasive lung adenocarcinoma patients with tumor size less than 3cm.

## INTRODUCTION

Lung cancer is one of the primary causes of cancer-related death in both men and women worldwide. [1] More and more early stage non-small cell lung cancer (NSCLC) have been detected recently due to rapid developments in imaging technology and widely application of thin-section computed tomography (CT) for screening high-risk patients [2, 3]. Adenocarcinoma is the most common type of NSCLC [4]. Despite early stage lung adenocarcinoma patients who had received curative-intent lobectomy, the post-surgical survival varies greatly. Visceral pleural invasion of lung cancer has been recognized as an adverse prognosis indicator for decades [5]. Previous studies indicated that VPI is an independent poor prognostic predictor regardless tumor size or N status. [6] However, there is no consensus on the impact of VPI on survival among patients with tumor size less than 3cm, especially less than 2cm [7]. In the 7th edition of the tumor, node, metastasis (TNM) classification system of lung cancer, pathological T stage upstages from T1a to T1b in the specimen of tumor size ≤3cm with VPI [8, 9]. Some recent researches demonstrated that for NSCLC patients with tumor size ≤3cm, there were no significant survival difference between patients with VPI or not [10-12]. In this study, we included a large cohort to evaluate the predictive value of VPI on survival among node-negative invasive lung adenocarcinoma patients with tumor size less than 3cm. Our result helps with management of early stage NSCLC patients with VPI.

## RESULTS

### Patient characteristics

**Figure 1:**
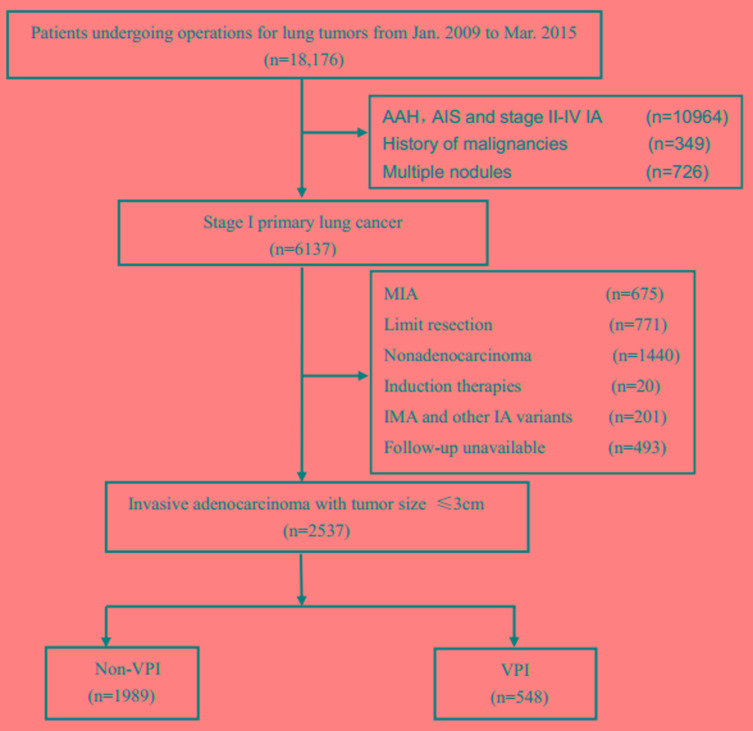
The selection process of eligible patients Abbreviations: AAH= Atypical adenomatous hyperplasia; AIS= adenocarcinoma *in situ*; MIA=microinvasive adenocarcinoma; IA=invasive adenocarcinoma; VPI= visceral pleural invasion

A total of 2537 patients were included in the cohort. Among them, there were 548 (21.6%) with VPI, 1989 (78.4%) without VPI. 1503 (59.2%) patients were females and 1034 (40.8%) patients were males. 1872 (73.8%) patients less than or equal to 65 years old, 665 (26.2%) patients older than 65 years old. Patients with tumor size less than or equal to 2cm account for the majority (1559, 61.5%), there were 978 (38.5%) patients with tumor size larger than 2cm but less than or equal to 3cm. Only 146 (5.8%) patients were detected with lymphovascular invasion. The number of patients with lepidic, acinar, papillary, micropapillary and solid were 226(8.9%), 1209(47.7%), 1002(39.7%), 13(0.5%), 87(3.4%) (Figure 1). The demographic features of 2537 patients were summarized in Table 1.

**Table 1:** Baseline characteristics of patients with stage I lung adenocarcinoma

Characteristic	Total (*N*=)2537	non-VPI (*n*=1989)	VPI (*n*=548)	p*
	No.	No. %	No. %	
**Sex**				.005
** Male**	1034(40.8)	782(39.3)	252(46.0)	
** Female**	1503(59.2)	1207(60.7)	296(54.0)	
**Age, years**				.132
** ≤65**	1872(73.8)	1499(75.4)	373(68.1)	
** >65**	665(26.2)	490(24.6)	175(31.9)	
**Tumor size(cm)**				.000
** ≤2**	1559(61.5)	1335(67.1)	224(40.9)	
** 2-3**	978(38.5)	654(32.9)	324(59.1)	
**Lymphovascular invasion**				.000
** Yes**	146(5.8)	85(4.3)	61(11.1)	
** No**	2391(94.2)	1904(95.7)	487(88.9)	
**ACT**				.000
**Yes**	500(19.7)	209(10.5)	291(53.1)	
**No**	2037(80.3)	1780(89.5)	257(46.9)	
**Adenocarcinoma subtype**				.000
**Lepidic**	226(8.9)	221(11.1)	5(0.9)	
**Acinar**	1209(47.7)	922(46.4)	287(52.4)	
**Papillary**	1002(39.7)	786(39.5)	216(39.4)	
**Micropapillary**	13(0.5)	8(0.4)	5(0.9)	
**Solid**	87(3.4)	52(2.6)	35(6.4)	
**TNM**				.000
**Ia1**	291(11.5)	291	0	
**Ia2**	1044(41.1)	1044	0	
**Ia3**	654(25.4)	654	0	
**Ib**	548(21.6)	0	548	

Abbreviations: ACT= adjuvant chemotherapy; VPI=visceral pleural invasion

*χ^2^ test was calculated from logistic regression model stratified by trail. *P* value is for the comparison between VPI and non-VPI.

### Survival outcome

**Figure 2:**
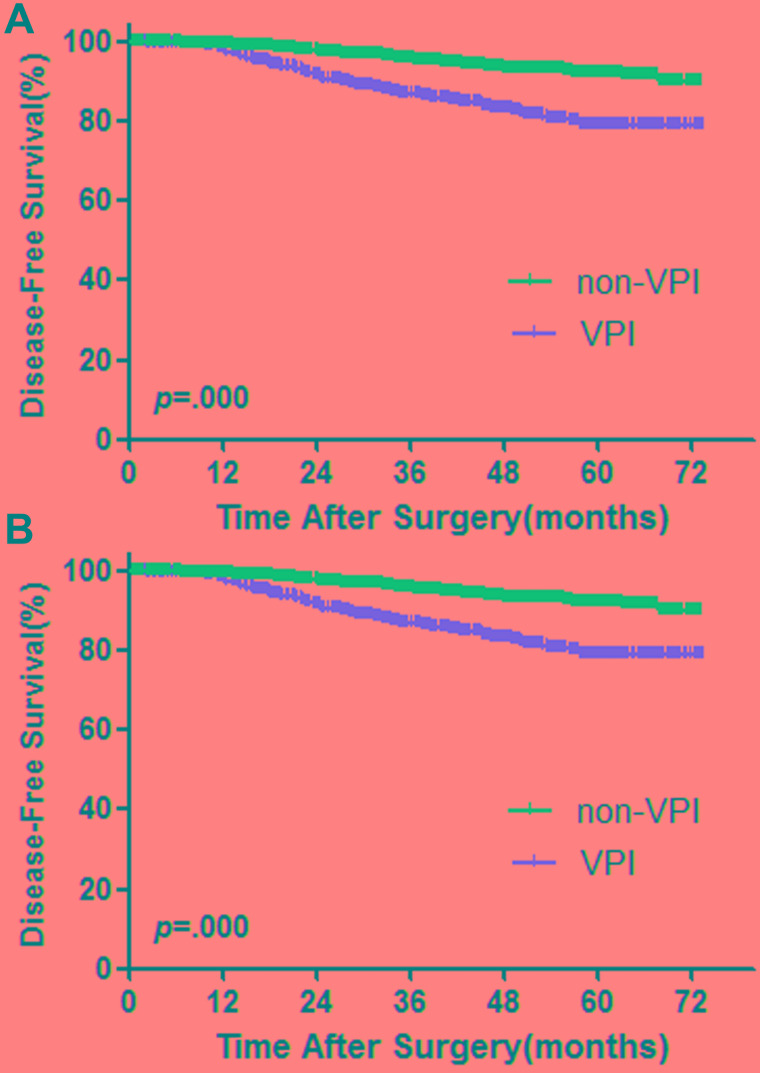
Survival cures for DFS (**A**) and OS (**B**) among patients with AIS, MIA and stage IA invasive adenocarcinoma**.** *p* values from log-rank test.

The median follow-up survival was 37.3 (3.0-87.63) months. Among the 2537 patients, 2371 (93.5%) were free of tumor recurrence and 166 (6.5%) developed recurrence. A total of 80 (3.2%) patients died during the follow-up period. First, we performed survival analysis in all 2537 patients. There were significant worse DFS and OS in patients with visceral pleural invasion (Figure 2).

**Figure 3:**
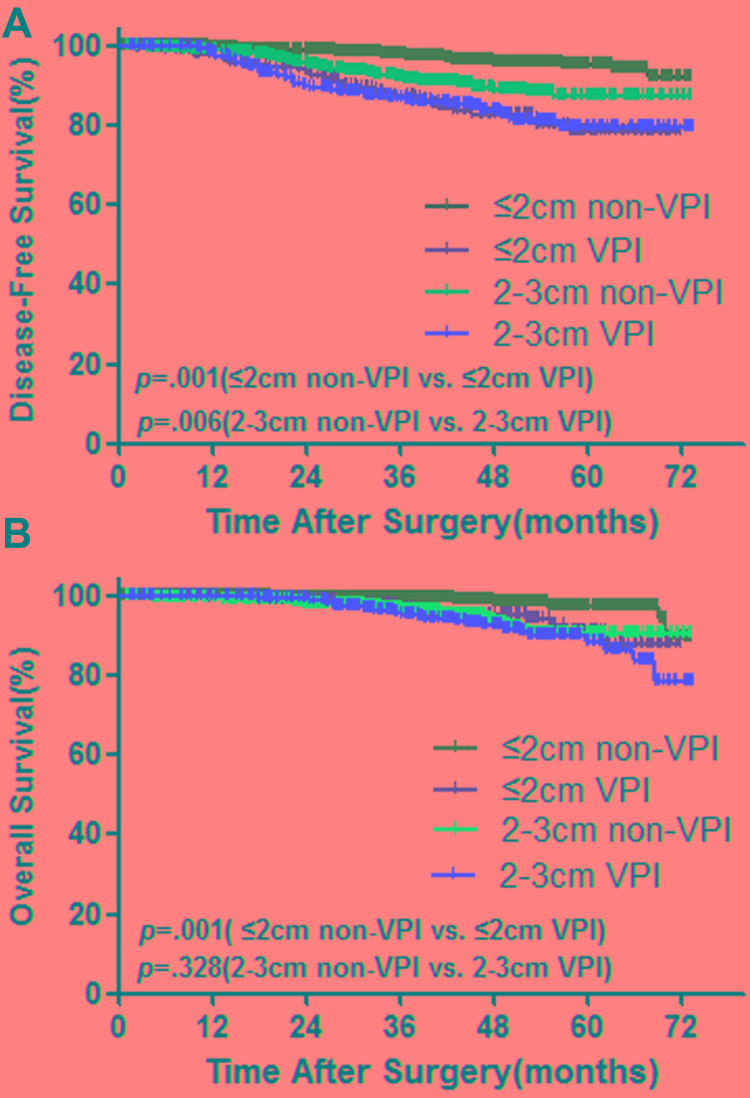
Survival cures for DFS (A) and OS (B) between patients with AIS/MIA and stage IA invasive adenocarcinoma *p* values from log-rank test.

In order to further evaluate the prognostic effect of VPI among patients with tumor size smaller than 3 cm, we next divided patients into 4 groups based on tumor size and visceral pleural invasion status. Group 1, tumor size ≤ 2cm and without VPI; group 2, tumor size ≤ 2cm and with VPI; group 3, tumor size > 2cm but ≤ 3cm, without VPI; group 4, tumor size > 2cm and ≤3cm and with VPI. The DFS and OS in each group were shown in Figure 3.

In tumor size ≤2cm groups, patients with VPI had significant worse DFS(HR,4.85; 95% CI, 2.98-7.91; *p* = .000) and OS(HR,3.52; 95% CI, 1.59-7.78; *p* = .002) compared with patients who had no VPI. In tumor size 2-3cm groups, patients with VPI had significant worse DFS (HR, 1.72; 95% CI, 1.16-2.55; *p* = .006), however, there were signifcant no difference for OS (HR, 1.31; 95% CI, 0.76-2.24; *p* = .330) compared with patients who had not VPI. For patients without VPI, tumor size was an independent predictive factor. Patients with tumor size 2-3cm had significant worse DFS(HR,2.96; 95% CI, 1.93-4.54; *p* = .000) and OS(HR,4.09; 95% CI, 2.18-7.67; *p* = .000) compared with patients with tumor size ≤2cm. However, at the presence of VPI, tumor size had no impact on survival. In patients with VPI group, there were no survival difference between tumor size 2-3cm group and ≤2cm group for both DFS(HR,1.02; 95% CI, 0.65-1.61; *p* = .939) and OS(HR,1.45; 95% CI, 0.71-2.97; *p* = .315).

Next, we performed multivariate analysis to determine independent prognostic and predictive factors for OS and DFS using Cox forward stepwise regression model. Sex, tumor size, histology subtype, LVI and VPI were found as independent prognostic factors for DFS. Sex, VPI and tumor size were found as independent prognostic factors for OS (Table 2).

**Table 2:** Multivariate analysis of overall survival and disease free survival

Predictor	DFS			OS		
	HR	95% CI	*P*	HR	95%CI	*P*
**Sex (male vs. female)**	1.61	1.18-2.19	.003.	2.59	1.62-4.14	.000
**Age (>60 vs.≤60)**	1.32	.97-1.80	.078	1.31	0.80-2.13	.268
**Visceral pleural invasion (Yes vs. No)**	2.37	1.70-3.31	.000	1.67	1.03-2.71	.039
**Lymphovascular invasion (Yes VS. No)**	2.33	1.53-3.56	.000	1.40	0.70-2.80	.338
**ACT (Yes vs. No)**	0.93	0.65-1.34	.699	0.88	0.52-1.50	.643
**Tumor size (2-3cm vs. ≤2cm)**	1.64	1.19-2.28	.003	2.57	1.57-4.20	.000
**Histology (SOL/MIP vs. LEP/ACN/PAP)**	2.19	1.32-3.62	.002	.046	2.09-4.31	.052

Abbreviations: ACT= adjuvant chemotherapy

## DISCUSSION

The adverse survival impact of VPI among early stage NSCLC patients remains controversial, especially in patients with tumor size less than 3cm [7, 13, 14]. This study, to our knowledge, collected the largest cohort for further evaluate the predictive value of VPI for post-surgical survival among lung invasive adenocarcinoma patients with tumor size less than or equal to 3cm. Our results indicated that, For patients with tumor size ≤3cm, VPI was a significant prognostic factor for poor survival regardless tumor size.

Although the 7^th^ TNM classification system had increased the T staging factor from T1 to T2a and upstages a tumor from stage IA to stage IB, it is still controversial whether VPI reduces DFS and OS among patients with invasive tumor size less than 3cm. David and colleagues [10] indicated that VPI was not strongly related to DFS or OS for tumor size <5cm. However, they did not present 5-year overall survival. A pool analysis [7] included more than 10 studies and examined the impact of VPI on 5-year OS with tumor size ≤3cm. The result demonstrated that patients with VPI had an unsatisfactory prognosis and regard VPI was an indicator of aggressive. Consistent with this pool analysis, our results showed that, among lymph node negative tumors ≤3cm, patients with VPI had a significantly worse survival in each tumor size group, and VPI was a major factor influencing survival rather than tumor size. A possible explanation for the results is visceral pleural is rich in lymphatic vessels, which eventually will join to the hilar lymph nodes [6, 15, 16]. As a consequence, VPI is associated with a higher frequency of locoregional recurrence and system metastasis. Post-surgical adjuvant chemotherapy may help to eliminate the residual cancer cells in the lymphatic vessel system. However, whether patients with VPI will benefit from adjuvant chemotherapy remains unclear. The current National Comprehensive Cancer Network (NCCN) guideline [17] suggests that patients with VPI should consider adjuvant chemotherapy. Unfortunately, this recommendation is lacking for high-level evidence support. Further clinical researches to figure the impact of adjuvant chemotherapy for VPI patients with tumor size ≤3cm are warranted.

According to the current NCCN guideline, for early stage NSCLC patients, the optimal therapy strategy is curative-intent surgical lobectomy with mediastinal lymph node dissection or systematic sampling [18]. Our previous studies demonstrated that, for patients with tumor size less than 3cm-segmentectomy could achieve comparable outcomes with lobectomy, and wedge resection offer equal outcomes with lobectomy only in some specific histological predominant patterns. In order to reduce the selection bias, we only included patients who had received lobectomy and systemic lymph node dissection.

Some limitations of our research should be mentioned. First of all, this is a retrospective study, patient selection bias is inevitable. Even though our study included a large cohort, however, the results should be further validated by multicenter cohorts. Second, the post-surgical follow-up was insufficient which may affect the accuracy of survival results. Third, not all patients with VPI had received adjuvant chemotherapy, which may influence the patients’ survival.

In conclusion, VPI is an aggressive prognostic factor for post-lobectomy survival for lymph node negative patients with tumor size less than 3cm. Our results are important for understanding biological behavior of VPI and help to guide the management of patients with VPI.

## MATERIALS AND METHODS

### Patient cohort

From January 2009 to March 2015, 18,176 consecutive patients with lung tumors undergoing resections in Shanghai Chest Hospital were identified. A total of 2537 patients with surgically resected pathologic N0 NSCLC with tumor size less than 3 cm in diameter were retrospectively reviewed. Correlative clinical data were retrieved from Shanghai Chest Hospital database. The study was approved by the Institutional Review Board of Shanghai Chest Hospital, Shanghai Jiao Tong University. All patients underwent complete resection of lung cancer. Inclusion criteria were invasive lung adenocarcinoma less than or equal to 3cm with available hematoxylin and eosin (H&E) slides for pathologic review. Exclusion criteria were adenocarcinoma in situ (AIS) and microinvasive adenocarcinoma (MIA), pathologic stage II disease and above, multicenter or metastatic disease, history of malignancy. Patients with invasion into the parietal pleura were excluded as well.

### Clinicopathological evaluation

Hematoxylin and eosin -stained (H&E) slides for each tumor were reviewed independently by two pathologists. The diagnosis of visceral pleural invasion is confirmed by elastic stain. The definition of visceral pleural invasion [19] is a tumor invades beyond the elastic layer with or without exposed on the pleural surface, but does not involve adjacent anatomic structures. Histopathologic criteria for AIS, MIA and invasive adenocarcinoma were according to the 2011 IASLC/ATS/ERS classification [20]. Pathologic TNM Staging was based on the 7th edition of the American Committee on Cancer (AJCC) cancer staging manual [21]. The clinicopathologic features including sex, age, tumor size, surgical procedure, lymphovascular invasion (LVI) and survival status were collected from patients’ medical records.

### Surveillance protocol

Overall survival (OS) was calculated as the number of months from pulmonary resection until the date of death. Disease-free survival (DFS) was calculated as the number of months from pulmonary resection until the date of diagnosis of recurrence. DFS and OS status were obtained from clinical medical records or telephone follow-up.

The postoperative surveillance protocol was described as our previous publications [22-24]: physical examination, chest CT, abdominal ultrasound examination was performed in every 6 months for the first year after surgery and at 6-month intervals thereafter. Whole-body bone scanning and brain magnetic resonance imaging (MRI) were performed once a year. Additional examinations were performed if patients had any symptoms occurred regardless of the follow-up schedule. For patients who did not follow-up in out hospital regularly, telephone follow-up were conducted to record the survival status.

### Statistical analysis

The χ^2^ test for categorical variables was used to compare patients’ characteristics between patients with VPI and without VPI. OS and DFS were estimated using the Kaplan-Meier method, and differences in survival were determined by log-rank analysis. Multivariable Cox regression analysis was used to assess the correlation of VPI with survival. Data were analyzed using Statistical Package for the Social Sciences Version 18.0 Software (SPSS Inc., Chicago, IL, USA). All *p* values were two-sided and p values less than 0.05 were considered statistically significant.

**CONFLICTS OF INTEREST**The authors declare no conflicts of interest.**GRANT SUPPORT**This article was supported by National Natural Science Foundation of China (81301996). The Ph.D Programs Foundation of Ministry of Education of China (20130101120017),Open Fund of Zhejiang Provincial Top Key Discipline of Pharmacology (YKFJ2-001), Medical Science and Technology of Heath of Zhejiang Provincial Government (2013KYA070).
